# Sequential packaging of RNA genomic segments during the assembly of Bluetongue virus

**DOI:** 10.1093/nar/gku1171

**Published:** 2014-11-26

**Authors:** Po-Yu Sung, Polly Roy

**Affiliations:** Department of Pathogen Molecular Biology, Faculty of Infectious and Tropical Diseases, London School of Hygiene and Tropical Medicine, Keppel Street, WC1E 7HT, UK

## Abstract

Bluetongue virus (BTV), a member of the Orbivirus genus within the *Reoviridae* family, has a genome of 10 double-stranded RNA segments, with three distinct size classes. Although the packaging of the viral genome is evidently highly specific such that every virus particle contains a set of 10 RNA segments, the order and mechanism of packaging are not understood. In this study we have combined the use of a cell-free *in vitro* assembly system with a novel RNA–RNA interaction assay to investigate the mechanism of single-stranded (ss) RNAs packaging during nascent capsid assembly. Exclusion of single or multiple ssRNA segments in the packaging reaction or their addition in different order significantly altered the outcome and suggested a particular role for the smallest segment, S10. Our data suggests that genome packaging probably initiates with the smallest segment which triggers RNA–RNA interaction with other smaller segments forming a complex network. Subsequently, the medium to larger size ssRNAs are recruited until the complete genome is packaging into the capsid. The untranslated regions of the smallest RNA segment, S10, is critical for the instigation of this process. We suggest that the selective packaging observed in BTV may also apply to other members of the *Reoviridae* family.

## INTRODUCTION

How virus genomes are packaged into their protective coats, or capsids, represents one of the foremost areas of virology where information is lacking. This is especially so for viruses with multipartite genomes, as a copy of each segment must be incorporated for the virus to be viable. The mechanism by which this is achieved in competition with the panoply of other nucleic acids present in the infected cell has proved elusive despite its critical importance to virus replication and survival. Bluetongue virus (BTV) is a complex, multi-layered, segmented double-stranded (ds) RNA virus and is the type member of the Orbiviruses, a genus in the family *Reoviridae*. As such it shares a virus family relationship with several other scientifically and medically important viruses (e.g. Rotaviruses). BTV is transmitted by insect vectors, replicating in both insect and mammalian cells, and can cause high morbidity and mortality in animals. BTV particles are non-enveloped, architecturally complex particles organized in two capsids. The outer capsid is composed of VP2 and VP5, which are responsible for virus entry in mammalian cells. The icosahedral inner capsid or core, with a diameter of 75 nm, is composed of two protein layers, the surface layer of 260 trimers of VP7 (38 kDa) which is built on a thin scaffold made up of 60 dimers of VP3 (100 kDa). The VP3 layer encloses a viral genome of 10 dsRNA segments of discrete sizes (S1–S10), together with the transcription complex of three proteins, VP1, VP4 and VP6 (1) termed the subcore. The genome is ∼19 kb in size, separated into 10 individual segments, S1–S10 (0.8–3.9 kb), which encode 7 structural (VP1-VP7) and 4 non-structural (NS1-4) viral proteins, each of which is involved in various stages of the virus replication cycle (2–6).

BTV enters mammalian cells via an endocytic pathway where the particle is uncoated (removal of VP2 and VP5) to release core particles into the cytosol. The genomic dsRNA segments are never released from the core; rather the intact core produces 10 capped messenger RNAs (mRNAs; single-stranded (ss) RNAs), one from each genomic segment, which are repeatedly synthesized by core-associated enzymes and extruded into the cytoplasm via pores in the 12 vertices of the icosahedral structure (7). These newly synthesized ssRNAs serve as mRNAs that express all viral proteins but they are also served as templates following packaging into newly formed viral cores where the synthesis of genomic dsRNAs subsequently takes place (8). In BTV-infected cells, ssRNA packaging and the assembly of cores occurs within virus-induced inclusion bodies, a matrix structure that is enriched with NS2 protein (9,10). NS2 has been shown to have sequence-specific affinity for BTV ssRNA segments and recruit BTV ssRNAs into the viral assembly location in BTV-infected cells (10–12). However, we have recently shown that NS2 is not necessary for *in vitro* ssRNA packaging (8).

Despite the considerable molecular and structural information available for BTV, the mechanism by which a complete set of 10 RNA segments is packaged remains unclear. Some segmented RNA genome viruses appear to have a non-selective packaging mechanism, for example, the two segments of infectious bursal disease virus genome are randomly packaged into virions and produce a large proportion of non-infectious particles with incomplete genome (13). However, it is mathematically impossible for the members of *Reoviridae*, containing 9–12 genome segments, to adopt this mechanism as the percentage of infectious particles containing complete genome would be too small. Moreover, the particles to infectivity ratio for these viruses is high (14–16), suggesting that there must be a selective packaging mechanism allowing the multiple genomic segments to correctly and effectively package into newly formed capsids.

The 10 segments of BTV vary both in sequence and size (from 0.8 to 3.95 kb) but are clustered in three distinct size classes (large, S1–S3; medium, S4–S6 and small, S7–S10). Broadly, two possible scenarios for packaging can be envisaged: each segment may contain a distinct packaging signal and only one of each is accommodated in the core; or packaging is restricted to only some segments but others are drawn in via their interaction with those that package, essentially ‘all-in’ or ‘follow-the-leader’ models of genome incorporation. To address these possibilities, it is essential to establish an appropriate assay system such as the recently established cell-free assembly (CFA) system for BTV which allows ssRNA packaging and capsid assembly *in vitro* (8). Each of the BTV inner core proteins was translated sequentially in an *in vitro* translation system together with 10 full-length positive sense ssRNA segments. These reconstituted cores were structurally and functionally identical to BTV cores (8). Here, using this system, we show for the first time that there is a packaging order for BTV ssRNA segments. We demonstrate that among all the BTV genomic segments, S10, the smallest segment with the longest untranslated regions (UTRs) (5′ UTR and 3′ UTR together = 155 bases) initiates RNA packaging. The other small genome segments also play a role in packaging, and the UTRs of S10 are essential for their involvement. In addition, using an *in vitro* RNA–RNA interaction assay system, we show that S10 plays a crucial role in first recruiting the other smaller RNA segments, probably forming a complex or complexes that then interact with larger segments. CFA confirmed this model as the basis for genome incorporation into nascent virus particles. We suggest that ordered RNA–RNA interactions are required for packaging the 10 RNA segments of BTV and that similar mechanisms may apply to other segmented dsRNA viruses.

## MATERIALS AND METHODS

### Plasmids and DNA templates

To generate T7 transcripts, template plasmids containing a T7 promoter and a specific restriction enzyme site flanking cDNA of exact copies of each BTV-1 genome segment (South African reference strain, Genbank accession numbers FJ969719–FJ969728), BTV-10 S10 (U.S. isolate, NC006015), AHSV-4 S10 (FJ183368) and Rhesus Rotavirus (RRV) S9 (EU636932.1) derived from viral dsRNA using the method of full-length amplification of cDNA (FLAC) were used. Chimeric S10 constructs were generated using 5′ primers encoding T7 promoter and 3′ primers (available upon request). A sequencing marker, replacing the sequence of 384–399 nt from 5′-GTTGAAAAGTGACCTA-3′ to 5′-ACTAAAGAGCGATTTG-3′ was also introduced in each chimeric S10 construct.

### Generation of T7 transcripts

Capped and uncapped ssRNAs were generated as previously described (17) using mMessage mMachine (Ambion) and T7 High yield Transcription RNA kits (Thermos), respectively.

### Cell-free *in vitro* assembly assay

Packaging of viral ssRNAs was investigated using a recently established CFA assay (8). Packaging efficiency was estimated using either ^32^P-labelled ssRNAs or non-radioactive quantitative reverse transcriptase-polymerase chain reaction (qRT-PCR). For ^32^P-labelled ssRNAs, T7 transcripts were 3′-end-labelled with 10 μCi 5′-^32^P-cytidine (Perkin-Elmer) using T4 RNA ligase (Fermentas). The CFA assay was carried out as described previously (8). Briefly, VP1, VP4, VP6, VP3 and VP7 were sequentially *in vitro* translated from capped ssRNA of coding regions, followed by incubation with full-length 10 BTV uncapped ssRNAs to allow viral core assembly. The whole mixture was loaded onto a continuous sucrose gradient and fractions were collected after ultracentrifugation. In the relevant fraction (fraction 6), unpackaged RNAs were eliminated by RNase One (Promega) digestion. Packaged RNA was extracted and analysed by denaturing agarose gel electrophoresis. To detect radiolabelled RNA, the gel was dried and exposed to a Storage Phosphor screen and analysed with Phosphor-imager and ImageQuantTL software (GE Healthcare).

### qRT-PCR

For detection of non-radioactive ssRNA, BTV-1 S6 or chimeric S10 were analysed by qRT-PCR using either primers reported by Toussaint *et al.* (18) or BTV-1 S10 335F: 5′-GTTGAAAAGTGACCTAGGAGGC-3′ and BTV-1 S10 492R: 5’-TTCACCACACCTAACATTGGG -3’, respectively. BTV RNAs from the packaging assay were RT into cDNA using ReverseAid Premium Reverse Transcriptase (Thermo) and quantified with suitable primers using 7500 Fast Real-Time PCR system and SYBR select Master Mix (Applied Biosystems). Three independent experiments were undertaken and qPCR was performed in duplicate. Standard deviations from the three experiments were calculated.

### *In vivo* packaging assay

10^6^ BSR cells were transfected as previously described (19) with 2 μg of uncapped T7 transcripts of wild-type or chimeric S10. The cells were subsequently infected with BTV-1 at a multiplicity of infection of 3. After 12 h, allowing for one replication cycle to be completed, cells were lysed and aliquots were stored for transfection control. Viral cores were then purified from the major portions of lysates as previously described. Unpackaged RNAs were digested with RNase at a final concentration of 1 μg/μl. Viral genomic RNA was then extracted, precipitated and subjected to qRT-PCR with a primer specific for the marker sequence (5′-ACTAAAGAGCGATTTG-3′) located in non-UTR or BTV-1 S10: BTV-1 S10 marker R: 5′-CCCAAATCGCTCTTTAG-3′. Copy number of marked S10 was correlated to the total BTV-1 S6 representing the number of total viral cores. Transfection discrepancy was further correlated with the copy number of marked S10 detected in the stored cell lysate aliquots.

### Reverse genetics (RG) system

To generate the virus with chimeric S10, BSR cells were firstly transfected with pCAG plasmid encoding primary replication complex (VP1, VP4 and VP6), VP3 and NS2 as described previously (17), followed by a second transfection with capped chimeric S10 ssRNA together with the remaining 9 BTV-1 ssRNAs. Replication of recovered viruses was visualized by plaque assay. To confirm the recovery of mutant virus, genomic dsRNAs were purified from the infected cells, reverse transcribed and the integrity of chimeric S10 was confirmed by nucleotide sequencing (Source Bioscience).

### RNA interaction assay

ssRNA of BTV-1 S10 was attached to beads leaving its 5′ and 3′ ends free by the following methods: streptavidin agarose beads (Novagen) were coated with a biotin-labelled primer which annealed to nt 401–430 in the coding region of S10 (5′-biotin-TTTTTTTTTTTGTATTATAGCTCTTTTCTTCTTTAAGCCTC-3′). The beads were incubated with poly-A RNA to decrease non-specific binding. BTV-1 S10 was then incubated with the coated beads followed by the addition of other ^32^P-labelled or non-labelled RNAs in an RNA folding buffer previously described (20). After 20 min incubation at 30°C, the beads were washed three times with excess folding buffer followed by 1 min heating at 90°C to release the RNA. For the radiolabelled RNA assay, samples were analysed by a denaturing gel and phosphor screen exposure. For non-labelled RNA, samples were analysed by qRT-PCR using primers specific for the target RNA, as described above. The S8- and S3-coated beads were similarly prepared using the biotin-labelled primers: 5′-biotin-TTTTTTTTTTGCTTCATCATCATCCAGCGTGACTCTTCCCTTGGC-3′ for S8 beads and 5′-biotin-TTTTTTTTTTCAACATCTATTGTAGCCCATCCATTATATCCTGTTCCTG-3′ for S3 beads.

## RESULTS

### The smaller BTV RNA segments initiate genome packaging

**Figure 1.**
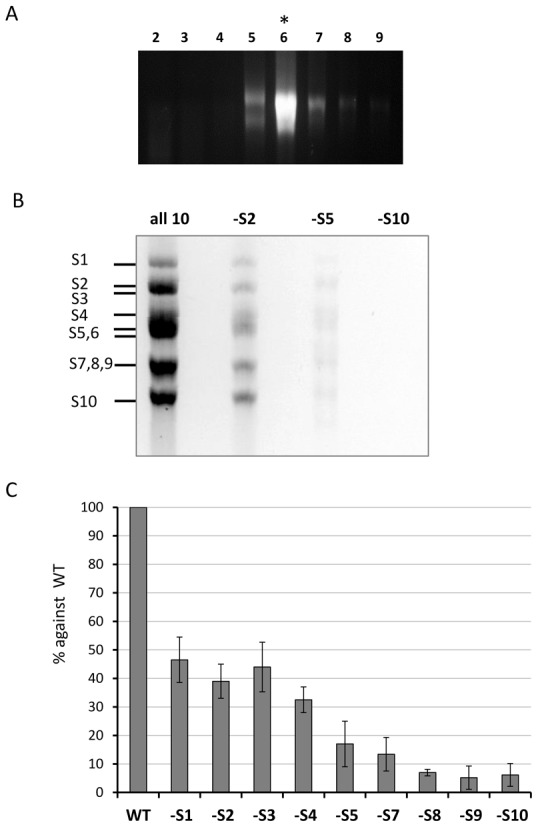
Exclusion of specific BTV RNA segment influences genome packaging. An incomplete set of ^32^P-labelled BTV ssRNAs that excludes S2 or S5 or S10 (indicated as -S2, -S5, -S10) but includes all the 9 respective segments were used in the *in vitro* CFA assay; the reaction mixture was purified on a sucrose gradient. A complete set of 10 ssRNAs was also included (all 10) as a control. (A) RNA distribution within the sucrose gradient fractions 2–9 was analysed on an agarose gel. The fraction containing assembled cores (fraction 6) is indicated with an asterix (*). (B) Packaged RNA profile after RNAse digestion and purification of fraction 6 analysed by 1% denaturing agarose gel. Segments S1–S10 are indicated on the left. (C) Quantification of the effect of segment exclusion. Ten BTV ssRNAs (WT) or ssRNAs excluding one ssRNA at a time (-S1, -S2, etc.) were used in the CFA assay. Packaged ssRNA in relevant fraction containing cores was purified on a sucrose gradient. BTV segment S6 was quantified by qRT-PCR to represent the packaging efficiency. Quantities of S6 in samples of -S1, -S2, etc. were compared with WT control in the same experiment and packaging efficiency was calculated. Standard deviations from three independent experiments are indicated (error bars).

To investigate if there is a preferential packaging of BTV genome segments, we sequentially excluded one RNA segment from mixtures of the full 10 RNA segments and used the recently developed *in vitro* CFA assay to determine the RNA packaging into the assembled core (8). Each of the BTV inner core proteins was translated sequentially in an *in vitro* translation system together with 10 full-length +ve sense ssRNA segments. To avoid any interference between protein translation and ssRNA packaging, only the coding region of each BTV transcript was used for translation assay while uncapped full-length ssRNAs were used for packaging. In brief, VP1, VP4 and VP6, the proteins that form the polymerase complex, were first generated individually using ORFs of S1, S4 and S9 segments, respectively, and then all three proteins were mixed and incubated with a set of ^32^P-labelled T7-driven 10 full-length BTV transcripts. For each experiment either a set of the complete 10 ssRNA segments, or a set of 9 segments excluding one large (S2), one medium (S5) or one small (S10) ssRNA segment was used. The reaction mixture was then incubated sequentially with *in vitro* expressed VP3 to form the subcore and VP7 to form a stable core structure. The newly assembled cores were purified by a sucrose gradient centrifugation and the fraction containing cores (fraction 6, Figure 1A (8)) was treated with RNase to remove unpackaged RNAs. The encapsidated RNAs in the cores were then phenol-chloroform extracted and analysed on a denaturing agarose gel. Figure 1B shows that when all 10 RNA transcripts were present, a complete set of BTV RNAs were resistant to RNase treatment, indicating that cores were synthesized and RNA packaged. When segment S2 was excluded, packaging of all segments was decreased while still apparent (∼40% compared to full set), when S5 was excluded, packaging was significantly reduced (∼10%), but when S10 was omitted RNA packaging was abolished (undetectable on Phosphor-imager). The experiment was performed in triplicate with the same result, indicating that omission of different RNA segments has a variable influence on RNA packaging and that S10 plays a critical role in the packaging of BTV ssRNA segments.

To further examine and quantify the effect of exclusion of each RNA segment, we undertook a further set of packaging experiments. Except for S6, all segments were excluded individually from the set of non-radioactive 10 ssRNA segments in the CFA assay. *In vitro* assembled cores were purified and treated with RNase as before. An aliquot of each sample was stored for protein analysis and packaging was measured using qRT-PCR for a marker BTV ssRNA, S6. The S6 packaging efficiency from each set of 9 ssRNA segments packaging versus the complete 10 ssRNA set of control experiments was assessed. The qRT-PCR comparison results demonstrated that when S1, S2 or S3 was excluded, the packaging efficiency was ∼50%, while the exclusion of S4, S5 or S7, the medium size ssRNAs, reduced packaging further (15∼30%); but most strikingly, the packaging efficiency was as little as 10% or less when any of the smaller segments (S8, S9 and S10) were excluded (Figure 1C). To confirm that protein expression in each experiment was equivalent, we determined the presence of polymerase complex protein VP6 and the major capsid protein VP7 by western blot analysis using mono-specific polyclonal antibody. All assembled samples, including controls and various segment exclusions, showed the presence of VP6 and VP7 proteins at similar levels, indicating that viral protein synthesis and core assembly was similar in all samples despite the absence of one segment (data not shown). The data confirmed a preferential role for smaller RNA segments in the initiation of BTV genome packaging.

### S10 UTRs influence BTV RNA packaging both *in vitro* and *in vivo*

**Figure 2.**
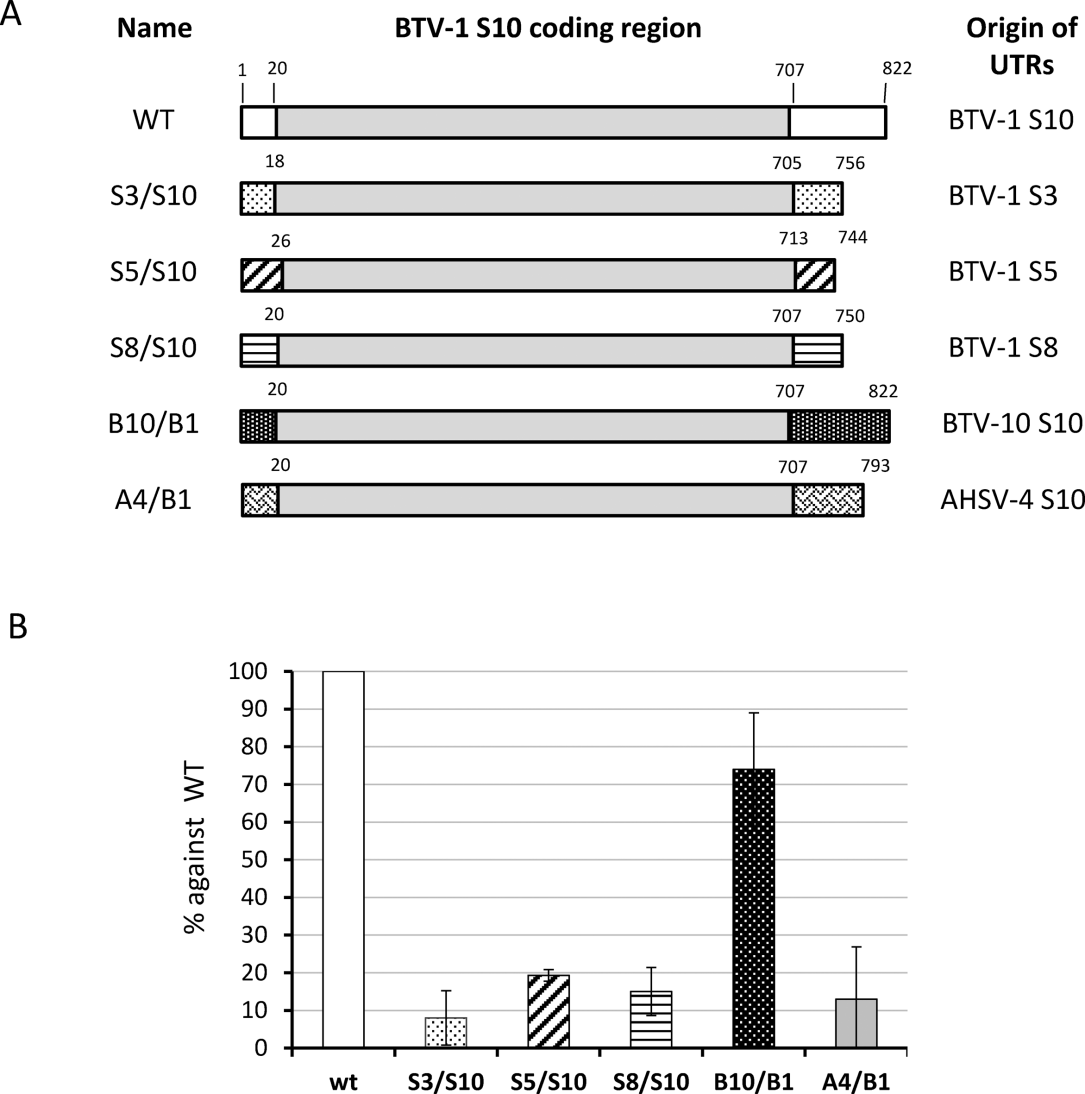
Exchanging UTRs of segment S10 influences packaging *in vitro*. (A) Schematic representation of chimeric segments S10. The UTRs of BTV-1 S10 (white boxes), flanking the open reading frame (grey boxes) were replaced with UTRs (pattern boxes) of BTV-1 S3 (S3/S10) or S5 (S5/S10) or S8 (S8/S10) or BTV-10 S10 (B10/B1) or AHSV-4 S10 (A4/B1) using overlap PCR and T7 transcription. (B) BTV-1 S10 (WT) or chimeric S10 (S3/S10, or S5/S10, or S8/S10, or B10/B1, or A4/B1) were included with segments S1–S9 in CFA assay. Packaged RNAs were purified and the encapsidation of S10 in each assay was measured by qRT-PCR using specific primers for the BTV-1 S10 coding region. The packaging efficiency and standard deviations (error bars) for each condition was calculated and normalized considering WT conditions as 100%.

As smaller segments appeared to play a critical role in BTV genome packaging, we investigated the relative roles of size and sequence identity. In many RNA viruses, specific packaging signals are mainly located in UTRs. Among the BTV RNA segments, S10 is the smallest (822 bases) but contains an unusually long 3′ UTR (118 bases) when compared to the UTRs of the other 9 RNA segments and is highly conserved in all serotypes. BTV cannot be recovered in the absence of S10 in a RG system despite the fact that deleting or replacing the majority of the coding region did not influence BTV replication suggesting that a cis acting RNA sequence, such as the UTRs, might be essential for viral replication (21). To verify if BTV S10 5′ and the long 3′ UTRs contain packaging signals, we designed chimeric ssRNA segments based on the coding region of BTV S10, with UTRs from different sources. To identify if S10 UTRs are essential for genomic RNA packaging, the UTRs of BTV-1 S10 were substituted with the UTRs of BTV-1 S3, S5 or S8, which are all different in both size and sequence. To verify the specificity of S10 UTR sequences, the UTRs of BTV-1 S10 were substituted with the UTRs of an alternate BTV serotype, BTV-10. The S10 of these two serotypes have similar but not identical sequences. In addition, S10 UTRs of a related orbivirus, African Horse Sickness Virus (AHSV), were also used to replace the UTRs of BTV-1 S10 (Figure 2A). The sequence and predicted structural differences among these UTRs are shown in Supplementary Figure S1. Each chimeric construct was confirmed by sequencing and subsequently utilized to synthesize chimeric ssRNAs by *in vitro* T7 transcription assay.

To determine the effects of altered UTRs on RNA packaging, each chimeric S10 together with the remaining 9 BTV-1 ssRNA transcripts were used in the CFA system described above. In parallel, wild-type S10 transcripts were used as a positive control. The chimeric S10 transcripts that were packaged into the newly constituted cores were quantified with qRT-PCR and the packaging efficiency compared to that of the control. When the BTV-1 S10 UTRs were substituted with the UTRs of S3, S5 or S8 of the same serotype, the packaging was significantly reduced in each case, indicating that the UTRs of S10 were important for S10 incorporation into the core. Similarly, changing the S10 UTRs of BTV-1 to the UTRs of AHSV-4 also reduced packaging substantially. However, replacement with S10 UTRs of an alternate BTV serotype (BTV-10), to BTV-1 UTRs, influenced the packaging only moderately (∼70% efficiency, Figure 2B). Thus, the packaging signals present in S10 UTRs are sequence-specific but closely related sequences from another BTV serotype could be tolerated.

**Figure 3.**
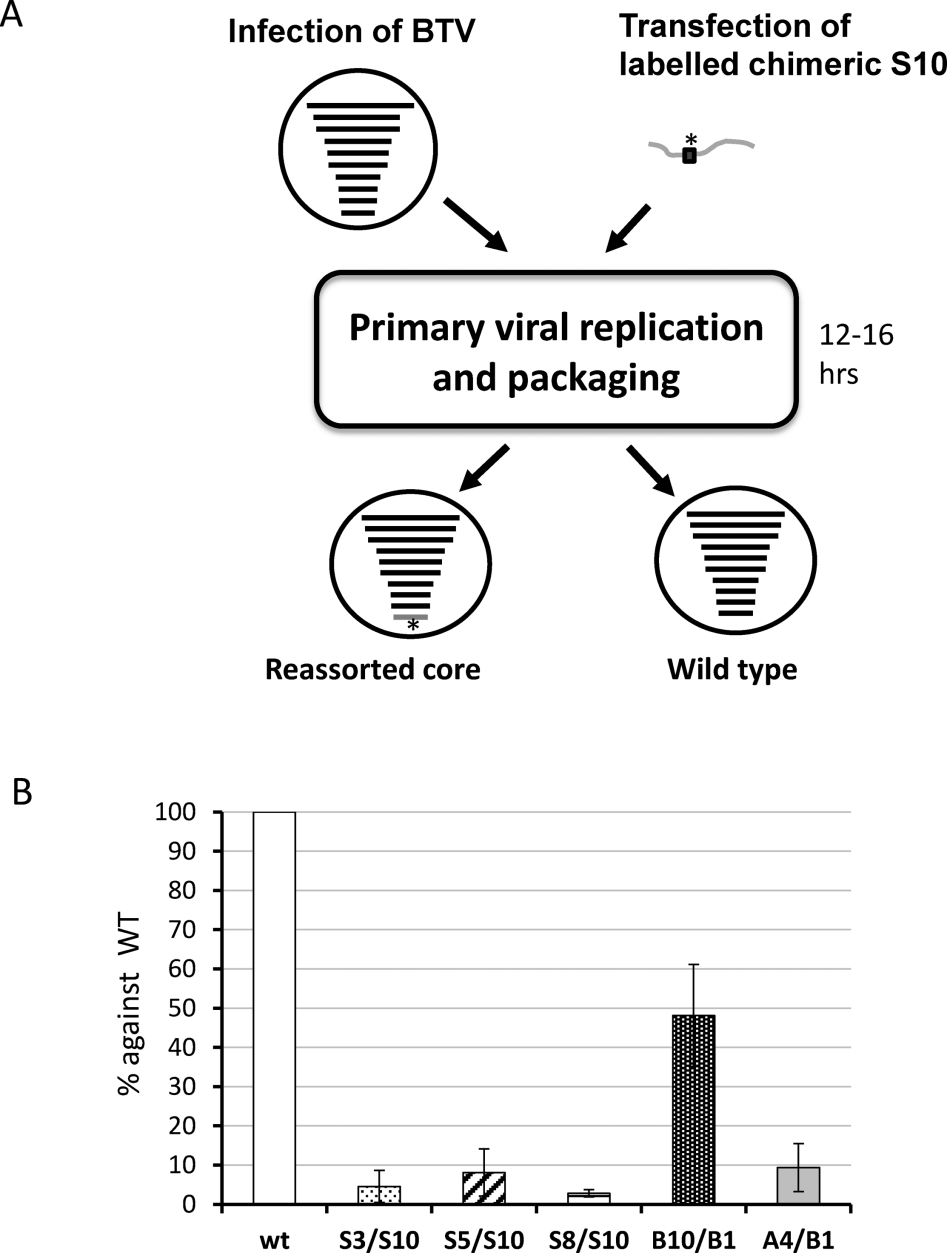
Effect of exchanging UTRs of S10 in packaging using an *in vivo* system. (A) A cartoon shows the process of *in vivo* single replication packaging assay. A modified sequence that can be specifically detected and quantified by PCR (marked with *) was introduced in the chimeric S10 ssRNAs used for transfection (in dark grey). Note that 12–16 h after transfection and infection, newly formed cores were purified and the amount of modified RNA packaged within the cores was measured. (B) Quantification of modified S10 (WT, S3/S10, S5/S10, S8/S10, B10/B1 and A4/B1) packaged in the new viral cores was correlated with the total quantity of new cores in the sample to obtain the packaging efficiency. The data was standardized to the wild-type data considered to be 100% and the ratios were calculated. Standard deviations are indicated as error bars.

To determine if this effect can also be reproduced *in vivo*, we adapted a recently established *in vivo* single-cycle packaging assay (19). The principle of this assay is that when BTV replicates in the cell, the progeny assembling cores will incorporate viral ssRNA segments from the cytoplasm. Therefore, if viral ssRNAs are transfected into cells prior to infection, both newly synthesized transcripts and transfected transcripts will be encapsidated. To perform the *in vivo* experiment, each chimeric S10 was introduced with a modified sequence in the coding region (nt 395, sufficiently distant to the UTRs) to facilitate specific detection and quantification by RT-PCR. This modification does not alter the amino acid sequence or the length of the segment (Figure 3A). BSR cells were transfected with each modified chimeric S10 transcript or wild-type S10 T7 transcript followed by infection with BTV-1. After 12–16 h post-infection, which allows for only one BTV replication cycle, transfected-infected cells were harvested and newly assembled viral cores were purified from the cell lysate as described (19). The modified S10 ssRNA packaged within the purified cores were then detected and quantified by qRT-PCR based on the specific modified sequence introduced in the S10 transcripts. To determine the packaging efficiency of the T7 ssRNAs, the copy number of the modified RNAs was correlated with the total number of transcripts present in the purified cores. The packaging efficiency of chimeric S10 was then compared with that of the control, wild-type S10 (Figure 3B). The BTV-1/BTV-10 chimeric S10 ssRNAs were found packaged into new viral cores with an efficiency of ∼50%, as expected for a transcript competing with endogenously produced wild-type BTV-1 S10. However, all other chimeric S10 ssRNAs (S3/S10, S5/S10 and S8/S10 chimeras, and BTV-1/AHSV-4 chimeras) packaged very poorly, if at all. These *in vivo* data are consistent with the *in vitro* data obtained from the CFA assay and confirms that S10 UTRs are essential for BTV genome segment packaging and that the packaging signals concerned are highly specific and located in the UTRs.

### Changing S10 UTRs blocked viral replication

**Figure 4.**
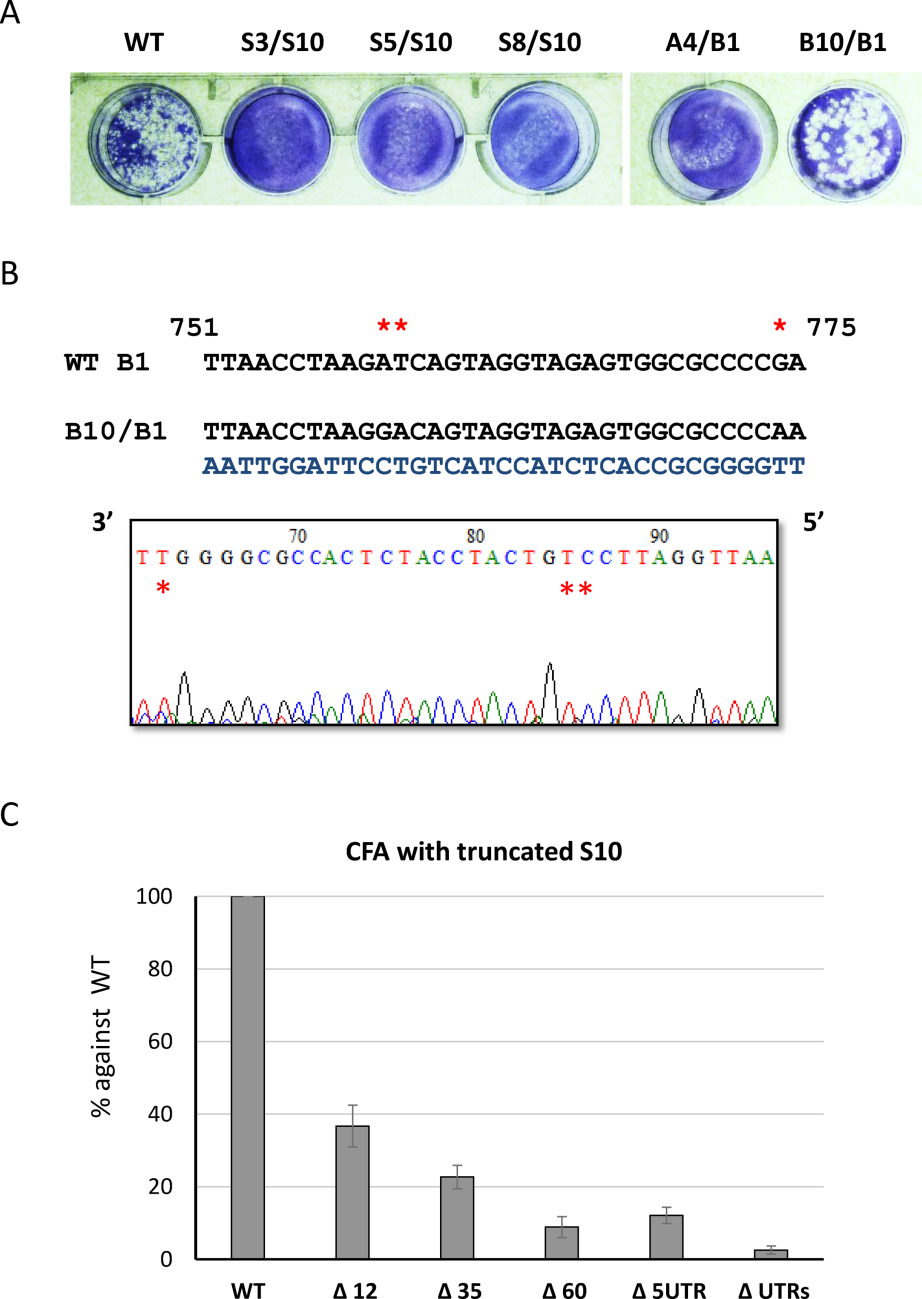
Effects of chimeric S10 on virus recovery using RG system. A complete set of 9 BTV-1 segments (S1–S9) and one of the chimeric S10 (S3/S10 to A4/B1) or the WT S10 were used in RG system. (A) Recovered viruses were amplified once and analysed by plaque assay on monolayers of BSR cells. (B) Genomic dsRNA of reassortant virus containing BTV-10/BTV-1 chimeric S10 (B10/B1) was purified and the sequence was confirmed by RT-PCR and sequencing. Nucleotides specific to BTV-10 in the electropherogram and in the actual sequence are indicated with an asterix. The sequencing data shows the reverse complement strand. (C) Deletion in UTRs suppresses packaging. BTV-1 S10 (WT), 5′ UTR and both UTRs truncated S10 (Δ5UTR and ΔUTRs) and a series of 3′-end truncated S10 (Δ12, Δ35, Δ60) were tested using CFA assay as described in Figure 2B.

The above studies demonstrate that changing the UTRs of S10 influenced packaging both *in vitro* and *in vivo*. To further determine if poor levels of packaging can be compensated in the cellular environment, we verified the effect of the chimeric S10 constructs on viral replication using a BTV RG system which allows for the introduction of an altered genome segment in a replicating virus (17). Accordingly, the five chimeric S10 constructs described above were introduced together with the remaining 9 other wild-type BTV segments using the RG system in an attempt to recover mutant viruses. Among the five chimeric mutants, only BTV carrying BTV-1/BTV-10 chimeric S10 was successfully recovered, as examined by plaque morphology and titres, in comparison to that of the wild-type virus (Figure 4A). That the recovered virus was not a revertant was confirmed by sequencing which showed the chimeric sequence to be present (Figure 4B). No virus was detected when the other four chimeric S10 RNAs were used despite multiple experiments (*N* = 3). Thus, changing the S10 UTRs by substituting with UTRs of other segments perturbs RNA packaging and effectively prevents viral replication. In contrast, when the S10 UTR is compatible with packaging, as in the BTV-1/BTV-10 exchange, packaging occurs and virus replication ensues.

Since S10 UTRs appear to be essential for BTV RNA packaging, we determined if certain specific region/regions in the UTRs are involved in RNA packaging. The 3′ terminal nucleotides of S10 was sequentially deleted from 12 to 60 nucleotides (12, 35 and 60) and each of these truncated S10 ssRNAs, together with remaining 9 full-length ssRNAs, were used for packaging in the CFA assay. When the packaging efficiency of each set was assessed, even the deletion of 12 nucleotides from the 3′ terminus suppressed packaging by more than 50%, and additional deletions further decreased packaging (Figure 4C). The data suggests that the end of S10 3′ UTR plays a significant role in BTV genome packaging. When the entire 5′ UTR or both 3′ and 5′ UTR of S10 was deleted there was essentially no packaging of the remaining ssRNA segments. These data indicate that both termini of S10 are important for packaging, probably via their interaction with each other. This is consistent with our previous data which showed that secondary structures instigated by the complementary sequences of the two termini play important role packaging of ssRNA segments (19).

### RNA–RNA networking is essential for packaging

**Figure 5.**
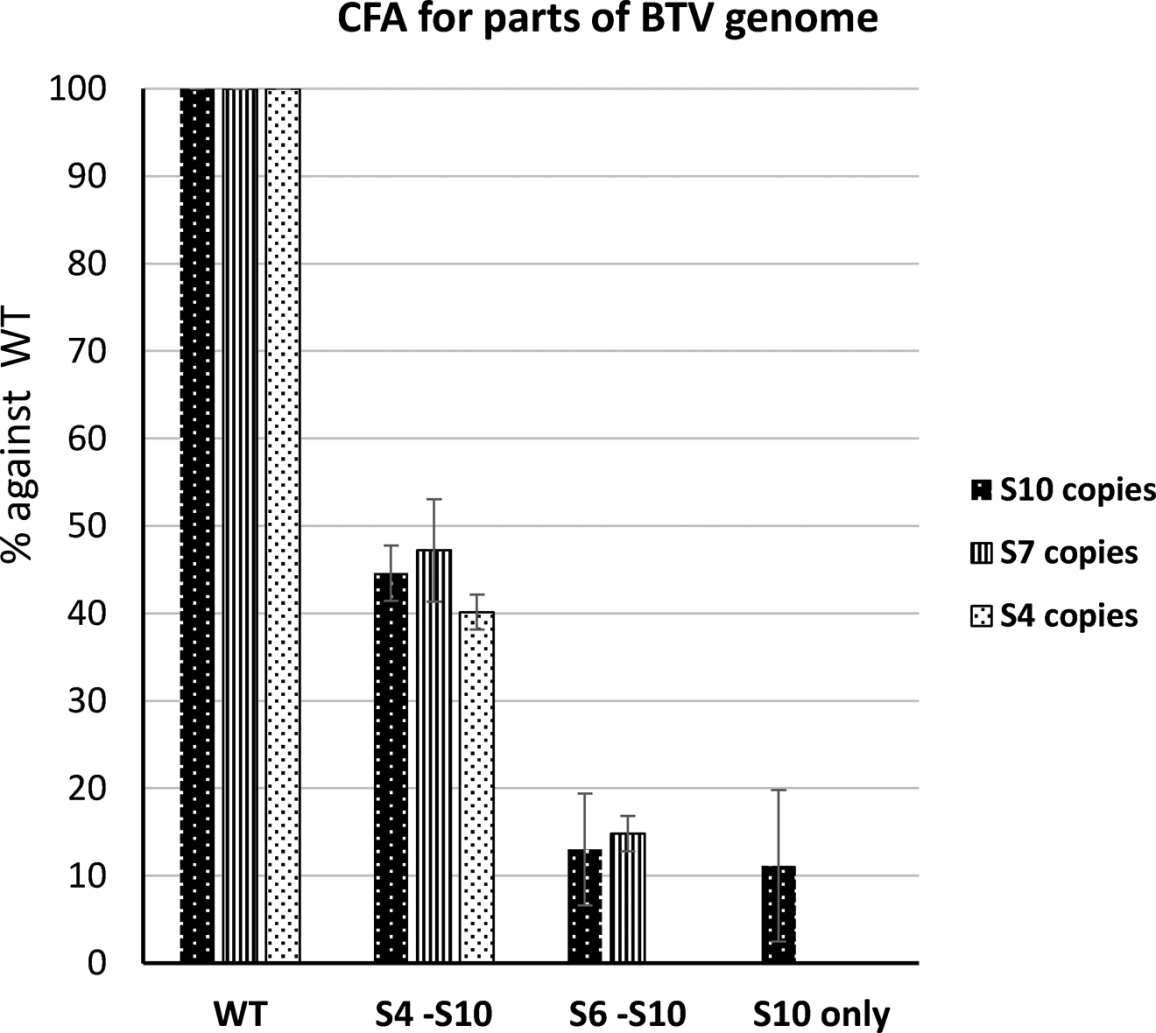
Complete set of BTV RNA segments is required for RNA packaging. Full set of BTV-1 10 ssRNAs (WT), S4–S10 (S4-S10), S6–S10 (S6-S10) or S10 only were used in CFA system to determine the packaging efficiency as described in Figures 1 and 2. Three segments (S4, S7 and S10; shown in different patterned bars as indicated) were detected with qRT-PCR when applicable. The packaging efficiencies are shown in percentage and standard deviation (error bars) were calculated.

The aforementioned data demonstrates that smaller segments are more important for BTV RNA packaging and that BTV RNA segments may form networks of size-related groups. Based on these, we hypothesized that such networking is important for BTV genome packaging. To demonstrate this, only certain genome segments were used in CFA system (Figure 5). Results obtained showed that although S10 was previously shown to be important for BTV RNA packaging and containing packaging signals, S10 alone was not packaged in this *in vitro* assembly system. Moreover, when S6–S10 RNAs were used for packaging in the absence of larger segments, packaging was substantially reduced when compared to packaging of the full set of 10 segments. However, the addition of S4 and S5 in the S6–S10 mixture increased the packaging 2-fold to an equivalent of the packaging efficiency when only one of the large segments (S1, or S2, or S3) was excluded as shown in Figure 1. In each packaging reaction, efficiency was determined by qRT-PCR analysis using three different segments (S4, S7 and S10) as necessary and all recorded similar incorporation. These results indicate that packaging of segments does not occur individually but rather depends on a complex formed by all RNA segments. It seems likely that a BTV genome was pre-assembled prior to being packaged into the capsid, and this assembly is based on a network of segments, which is initiated by the smaller segments. Therefore, although S10 appears to be the critical segment for initiating the network, it is not sufficient for packaging in the absence of other smaller segments.

### S10 interacts with other BTV RNA segments

**Figure 6.**
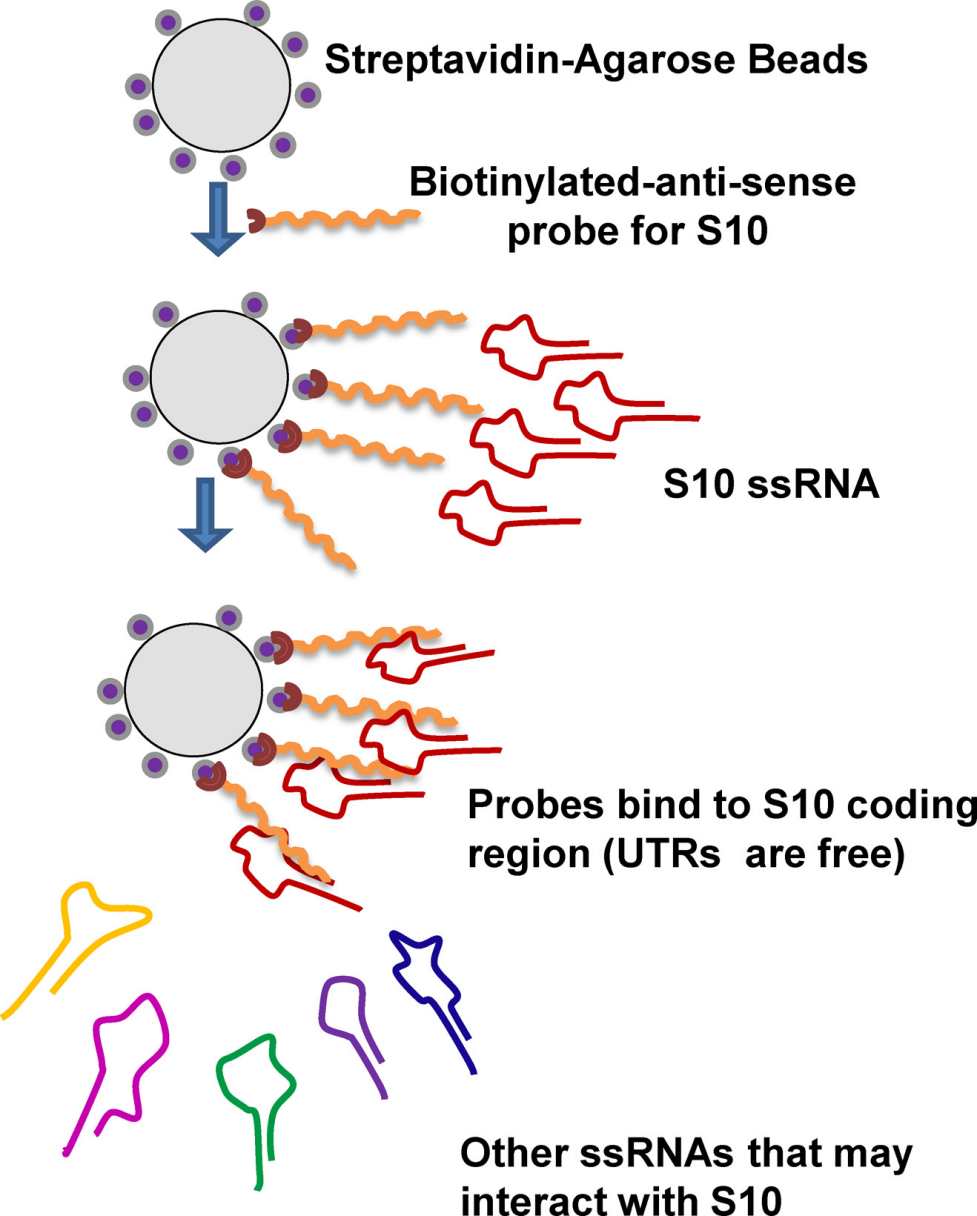
A schematic for RNA–RNA interaction assay based on BTV S10-coated beads.

**Figure 7.**
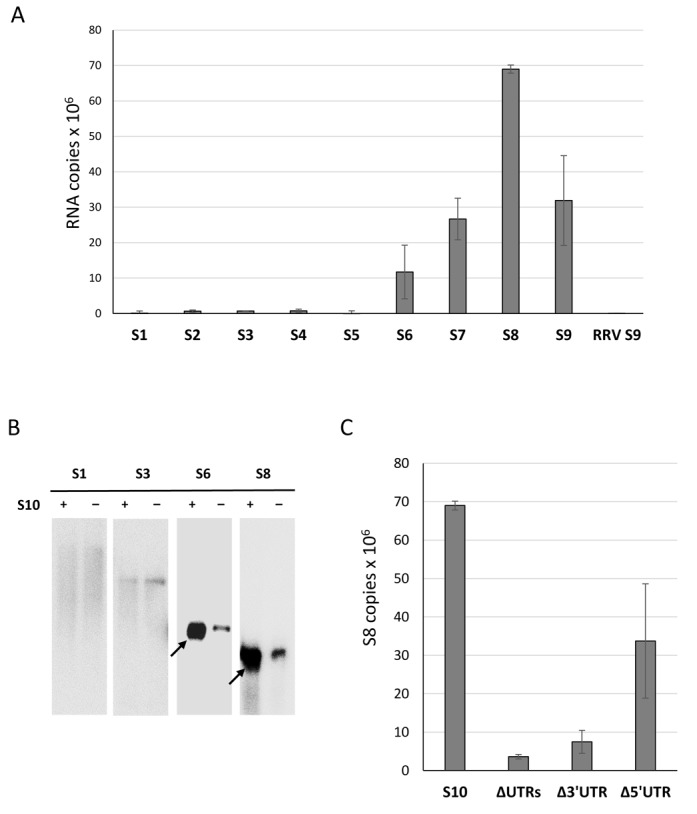
BTV S10 interacts with smaller segments. (A) Beads coated with a S10-specific primer were incubated sequentially with BTV-1 S10 and with 1 pmol of segments S1–S9 individually. Interaction with RRV S9 was included as a negative control. After extensive washing, the attached RNA was released by heating. The amount of interacting RNA was determined by qRT-PCR using primers specific to each segment. The copy number was correlated by minus non-specific binding detected in beads-only control. S10 and the standard deviations from three individual experiments are indicated. (B) Beads coated with (+) or without (−) BTV-1 S10 were similarly prepared and sequentially incubated with ^32^P-labelled S1, S3, S6 or S8. After three washes, RNAs were heat-released and analysed on a denaturing agarose gel and Phospho-imager exposure. The black arrows indicate positive interaction. (C) The interaction between S8 and truncated S10 was measured with a similar method: beads were coated with BTV-1 S10 (S10), S10 lacking 5′ and 3′ UTRs (ΔUTRs), 3′ UTR (Δ3′UTR) or 5′ UTR (Δ5′UTR) and incubated with equal amounts of BTV-1 S8. Interacting S8 was analysed and quantified similarly. Interaction rates and standard deviation (error bars) were calculated.

As the smallest BTV RNA segment, S10, appears to initiate the packaging of the remaining RNA segments, we investigated if S10 RNA recruits other segments by direct interaction. To detect interactions between different RNA segments, we designed a primer binding assay based on streptavidin beads as shown in a schematic (Figure 6). Since the UTRs of S10 were important for assembly, it was necessary to keep both the 5′ and 3′ termini free, unbound to beads. A biotinylated primer which specifically binds to the centre of the S10 coding region was designed. The primer was used to coat the streptavidin beads and allowed to anneal to the S10 RNA. Then, different BTV RNA segments (BTV S1–S9 and ssRNA of a non-related RRV RNA, S9), each at 1 pmole, were incubated with the coated beads. After washing, the attached RNA from each reaction was released and detected by qRT-PCR using segment-specific primers. Non-coated beads served as the negative control. The results indicated that S10 had a high affinity for the small BTV segments, S7, S8 and S9, particularly S8 and a moderate affinity for S6, a medium size RNA segment of BTV (Figure 7A). S10 did not interact with the larger segments and showed essentially no affinity for RRV RNA S9 (814 bases). This suggests that the interaction between S10 and smaller BTV segments is not due to their size but is more likely sequence or secondary structure specific. To confirm these results, the same RNA–RNA pull-downs were also performed using radiolabelled ssRNAs. ^32^P-labelled S1, S3, S6 and S8 were incubated separately with beads coated with unlabelled S10 RNAs as described above. Beads not coated with S10 were used as controls. After extensive washing, the bound ^32^P-RNAs were released from the beads by heating at 90°C and analysed on a denaturing agarose gel followed by autoradiography. It was clear that while both S6 and S8 had interacted with S10, the larger segments S1 and S3 failed to bind S10 (Figure 7B). As the S10 UTRs are important for packaging, they plausibly also play a role in RNA–RNA interaction. To verify this, primer bound beads were coated with the coding region of S10, S10 with the 3′ UTR or S10 with the 5′ UTR only. Coated beads were incubated with S8, a representative segment shown to have the highest affinity for wild-type S10, and the binding was estimated. When both UTRs were removed, S10 largely lost its affinity for S8 and this was also the case when the 3′ UTR was removed. However, when the 20 bases of 5′ UTR were removed, S10 and S8 interacted to a level of ∼50% of the parental molecule (Figure 7C). Thus, the 3′ UTR of S10 is critical for the observed S10–S8 interaction while the 5′ UTR is not essential but might enhance it.

### Smaller segments can act as intermediates for binding the larger segments

**Figure 8.**
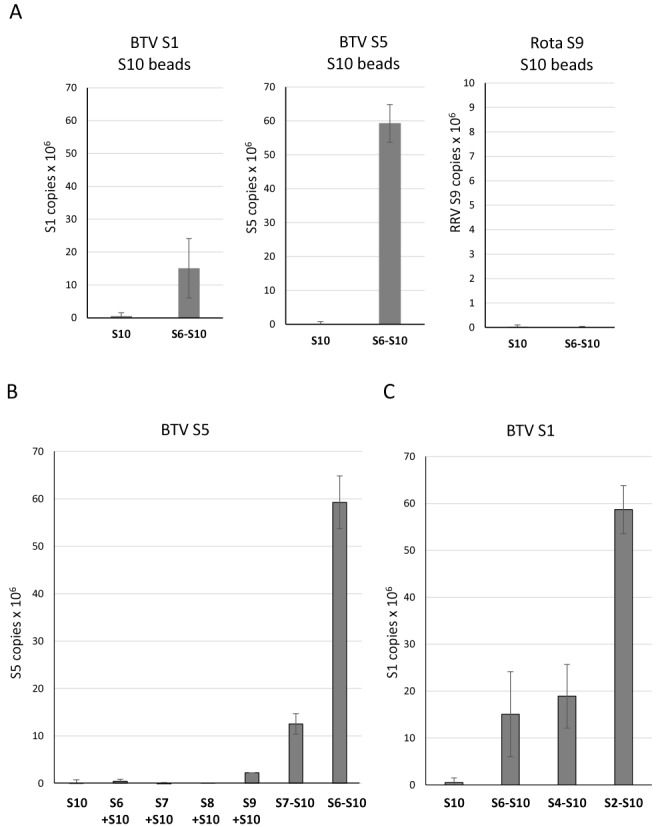
Smaller segments link larger segments to S10 in a specific order. S10-coated beads were prepared as described in Figure 6. (A) 1 pmol BTV-1 S1, S5 or RRV S9 were incubated with S10 beads only (S10) or together with a mixture of S6, S7, S8 and S9 (S6-S10). Interacting ssRNAs were analysed by qRT-PCR and correlated to the control lacking S10. Standard deviations from three individual experiments are indicated (error bars). (B) Enhancement of the interaction between S5 and S10. BTV-1 S5 was incubated with S10 beads alone (S10) or with S6, S7, S8 or S9 separately (S10 + S6, etc.) or with a mixture of S7 to S9 (S10 + S7-S9) or S6 to S9 (S10 + S6-S9). (C) The interaction between S1 and S10 was enhanced when adding a different mixture of segments. BTV-1 S1 was incubated with S10 beads alone (S10) or with a mixture containing S6 to S9 (S6-S10), or S4 to S9 (S4-S10), or S2 to S9 (S2-S10).

Isolated S10 exhibited an affinity *in vitro* for the smaller BTV segments but not the medium or large segments. However, for BTV genome packaging, all 10 segments have to be included to form a complete genome set. To enable this, the smaller segments plausibly form a complex which is then linked to other segments. To verify this hypothesis, we added S6, S7, S8 and S9 onto the S10 beads followed by incubation of the mixture with S1 or S5, as representatives of large and medium size segments, respectively, each of which previously failed to bind to S10 directly. Clearly, in the presence of other small segments, both S1 and S5 were successfully pulled-down but not by S10 alone, while there was no change for the RRV RNA control (Figure 8A). These data indicate that a complex might have formed with S6–S10, which was probably necessary to pull-down the larger segments.

Further, we investigated whether the increased affinity was mediated by one or more specific segments of the four RNA segments, S6–S9, or if all RNA segments formed a complex to recruit larger segments. When each of the small segments, S6–S9 was added separately into the interaction assay of S10 with S5, only S9 slightly enhanced the interaction (Figure 8B). However, a combination of three small segments (S7–S10) significantly enhanced interaction with S5. Moreover, the presence of S6 in this mixture increased the affinity 5-fold. None of the individual segments alone affected the S5 and S10 interaction, only a mixture of segments affected the binding of S5, suggesting that it is essential that smaller segments form a complex to bind to S5. When confirming this with S1, it was clear that the mixture containing S4–S9 had a similar effect to the one seen with the S6–S10 and the binding of S1 did not improve. However, when S2 and S3 were added to the mixture, the affinity increased 3-fold (Figure 8C). These interactions suggest that there is an order of BTV genome RNA packaging, at least in the *in vitro* packaging assay, and that smaller segments form a complex which binds medium and then larger segments to effect full genome encapsidation.

**Figure 9.**
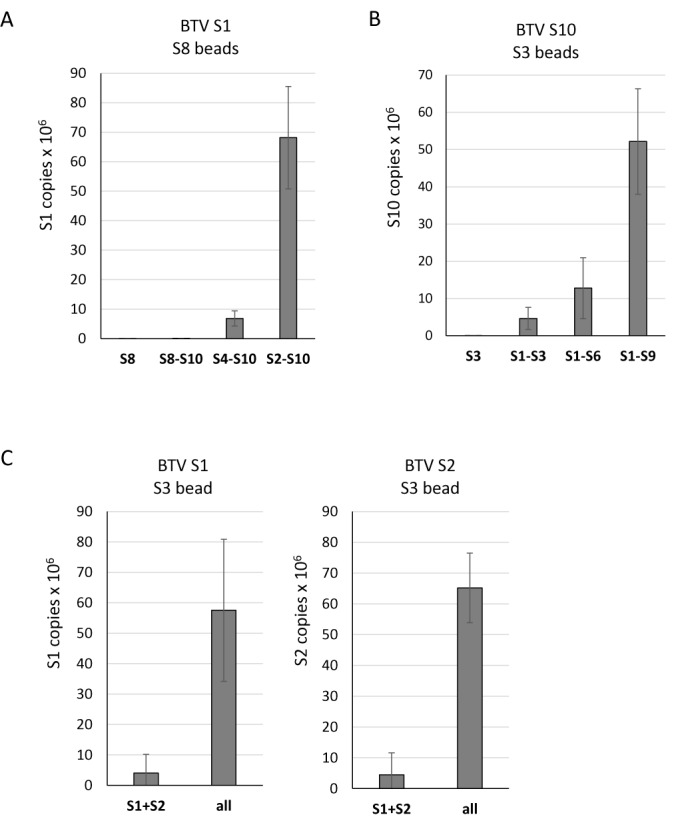
Specific interaction order among segments with different sizes. (A) Smaller segments link larger segment S1–S8. S8-coated beads were prepared similarly as S10-coated beads as described. BTV-1 S1 was incubated with S8 beads alone (S8) or with mixtures of S8 to S10 (S8-S10), or S4 to S10 (S4-S10), or S2 to S10 (S2-S10). The pulled-down S1 was quantified with qRT-PCR as described. (B) Larger segments also link smaller segment S10 to S3. S3-coated beads were similarly prepared. BTV-1 S10 was incubated with S3 beads alone (S10) or with different mixtures (S1-S3, or S1-S6, or S1-S9). (C) S3 beads were incubated with BTV-1 S1 and S2 or with all other 9 segments. The pulled-down S1 and S2 were quantified as described. The increased interaction was shown in bar and standard deviation (error bars) were calculated.

This was substantiated further by using an alternate smaller segment, S8, instead of S10, where the data obtained was similar (Figure 9A). There was no interaction when only S1 was added. Furthermore, S1 was not pulled-down by the complex formed between S8 and other smaller RNA segments (S9, S10). However, the mixture of S4–S10 interacted better with S1 and when S2 and S3 were added, the recruitment of S1 increased 5-fold. In contrast, when S3 beads, instead of S8, were used to pull-down S10, there was no interaction and also significantly less when only large and medium size ssRNAs were used. S10 was pulled-down most efficiently when all small segments were present (Figure 9B). To clarify if a BTV RNA segment complex was initiated preferentially by the smaller segments as suggested by data in Figure 1, we further analysed the interaction between S3 and other larger segments (S1 and S2). S1 and S2 alone did not exhibit strong interaction with S3 beads (Figure 9C). In contrast, when all 10 segments were present, S1 and S2 pull-down were increased. This data confirmed that an RNA–RNA network results in correct BTV genome packaging and that it is initiated by the smaller segments.

## DISCUSSION

Much effort has been devoted to investigating how segmented genome RNA viruses correctly incorporate their RNA genome. There are two possible strategies. First, for viruses, such as influenza virus (IFV), the RNA genome assembles first and the viral capsid forms surround these genomes. The eight segments of IFV replicate separately and are encapsidated into ribonucleoprotein structures (22). The segments are then packaged into new virions. The current understanding is that this packaging is a selective rather than a random process (23–25). Various sequences were found to serve as packaging signals in both coding and non-coding regions of IFV genome (26–28). It has also been shown that the 8 IFV RNA segments have intermolecular interaction which is essential for IFV genome packaging (26,29,30). Second, for some dsRNA viruses, such as bacteriophage phi 6 and 12, positive strand ssRNAs are first actively transported into a ready-formed capsid and are then converted into dsRNA genomic segments by the resident polymerase. In this case, the three segments of RNA genome are recognized sequentially by the procapsid, especially an ATPase protein, P4, which serves as a packaging motor protein (31–33).

The data described in this report suggests that BTV, and possibly other members of the *Reoviridae* family, follow the first strategy: the ssRNA segments of the viral genome assemble prior to packaging as an RNA complex orchestrated by the smallest segment, S10. The core of BTV and other members of the family is a highly rigid icosahedral structure with 12 pores, one in each 5-fold vertex, through which the newly synthesized viral positive strand ssRNAs extrude (34). Our recent data has shown that transcripts of each segment extrude through a specific pore and that it is not a random process (35). For the reverse process, i.e. the packaging of ssRNA through these narrow channels, RNA segment entry would have to occur one at a time, making it difficult to explain how excluding a single segment influences the packaging of other segments. Our data, obtained from both *in vitro* packaging and RNA–RNA interaction studies, suggests that the BTV genome segments assemble first through the formation of a network. The network of complete set of genome segments is then packaged into the inner capsid layer formed by VP3 although it cannot be ruled out that the BTV genome and VP3 capsid co-assemble, as the interaction between RNA genome and VP3 may have a role in correct packaging.

Our data suggesting a packaging order for different segments of the BTV genome is not unprecedented: the three-segmented phi 6 phage packages the RNA segments in order of small to large through specific packaging signals (36–39). The packaging order for 10 segments of BTV, each with different sizes and sequences, however, is likely to be more complex. Our data strongly suggests that S10, especially its UTRs region, play a crucial function in RNA packaging and it is likely that interaction of the 5′ and 3′ UTRs drive the formation of secondary structure of S10 ssRNAs, necessary for recruiting other ssRNAs. However, other small segments (S7, S8 and S9) also interact with S10 to trigger the assembly of all 10 ssRNAs. For example, S8 beads needed medium and larger segments to pull-down S1, similar to that of S10 beads. The data rationalizes this finding by showing that the smaller segments form a complex, which then recruits the other segments. In this sense, each smaller segment (S7–S10) might be equally important for BTV RNA packaging, despite the fact that the longer 3′ UTR of S10, which is conserved among serotypes, enables it to play a more key role in assembly. Interestingly, although the largest segment, S1, was captured by the small segment complex the interaction was enhanced significantly when large segments S2 and S3 were added. This data suggests that the 10 BTV segments may form several complexes which combine to result in a form compatible for packaging. Moreover, although S10 is crucial to BTV RNA packaging, neither S10 alone nor S10 plus other smaller segments were packaged efficiently. Only when larger segments were included were all segments equally packaged. This is consistent with an ‘all-in’ genome incorporation model despite the fact that RNA–RNA interactions adopt a ‘follow-the-leader’ model to assemble the packaging complex. Further studies on the RNA interactions among the BTV segments are needed to clarify how each segment separately interacts with each other to form such a chain of complex structures.

Our data indicates that the UTRs of S10 are critical for BTV assembly through a sequence-specific or secondary structure-specific mechanism. Even a short deletion (12 NT) from the 3′ terminus of S10 perturbed the packaging of the ssRNAs during assembly. Alignment of the different BTV serotypes shows a high level (over 80%) of conservation in the unusually long S10 UTR (shown in Supplementary figure). It is possible that S10 interacts with other segments in some of these regions, which is consistent with the model that RNA secondary structure serves as the genome packaging signal for segmented viruses (40–42). Our recent study also concluded that BTV segments contain structural signals for packaging (19). A combination of functional analysis and direct probing of RNA structure would be required to reveal the actual structures of the viral RNA segments concerned (40,43–45).

## SUPPLEMENTARY DATA

Supplementary Data are available at NAR Online.

SUPPLEMENTARY DATA
