# Supplementary material for: Sequential packaging of RNA genomic segments during the assembly of Bluetongue virus
Source: Nucleic Acids Res. 2014 Nov 26;42(22):13824–38. doi: 10.1093/nar/gku1171 (PMC4267631; doi:10.1093/nar/gku1171)
Supplement: SUPPLEMENTARY DATA [file supp_42_22_13824__index.html]

Sequential packaging of RNA genomic segments during the assembly of Bluetongue virus — Sequential packaging of RNA genomic segments during the assembly of Bluetongue virus — SUPPLEMENTARY DATA 

# Sequential packaging of RNA genomic segments during the assembly of Bluetongue virus

## SUPPLEMENTARY DATA

**Files in this Data Supplement:**

- SUPPLEMENTARY DATA
