# Supplementary material for: Sequential packaging of RNA genomic segments during the assembly of Bluetongue virus
Source: Nucleic Acids Res. 2014 Nov 26;42(22):13824–38. doi: 10.1093/nar/gku1171 (PMC4267631; doi:10.1093/nar/gku1171)
Supplement: SUPPLEMENTARY DATA [file supp_gku1171_nar-01925-r-2014-File011.pptx]

## Slide 1
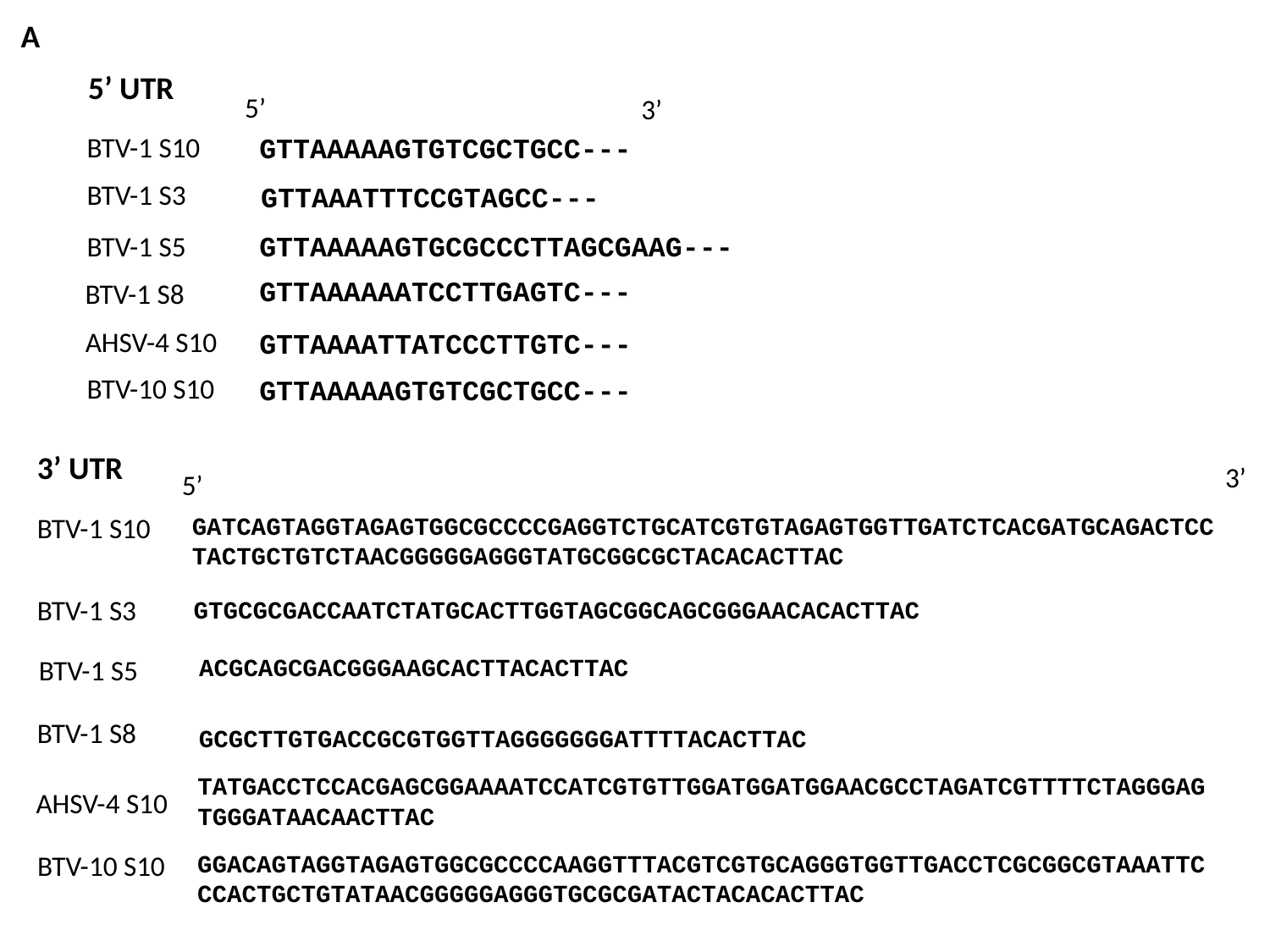

A
5’ UTR
5’
3’
BTV-1 S10
GTTAAAAAGTGTCGCTGCC---
BTV-1 S3
GTTAAATTTCCGTAGCC---
GTTAAAAAGTGCGCCCTTAGCGAAG---
BTV-1 S5
GTTAAAAAATCCTTGAGTC---
BTV-1 S8
AHSV-4 S10
GTTAAAATTATCCCTTGTC---
BTV-10 S10
GTTAAAAAGTGTCGCTGCC---
3’ UTR
3’
5’
BTV-1 S10
GATCAGTAGGTAGAGTGGCGCCCCGAGGTCTGCATCGTGTAGAGTGGTTGATCTCACGATGCAGACTCCTACTGCTGTCTAACGGGGGAGGGTATGCGGCGCTACACACTTAC
BTV-1 S3
GTGCGCGACCAATCTATGCACTTGGTAGCGGCAGCGGGAACACACTTAC
ACGCAGCGACGGGAAGCACTTACACTTAC
BTV-1 S5
BTV-1 S8
GCGCTTGTGACCGCGTGGTTAGGGGGGGATTTTACACTTAC
TATGACCTCCACGAGCGGAAAATCCATCGTGTTGGATGGATGGAACGCCTAGATCGTTTTCTAGGGAGTGGGATAACAACTTAC
AHSV-4 S10
BTV-10 S10
GGACAGTAGGTAGAGTGGCGCCCCAAGGTTTACGTCGTGCAGGGTGGTTGACCTCGCGGCGTAAATTCCCACTGCTGTATAACGGGGGAGGGTGCGCGATACTACACACTTAC

## Slide 2
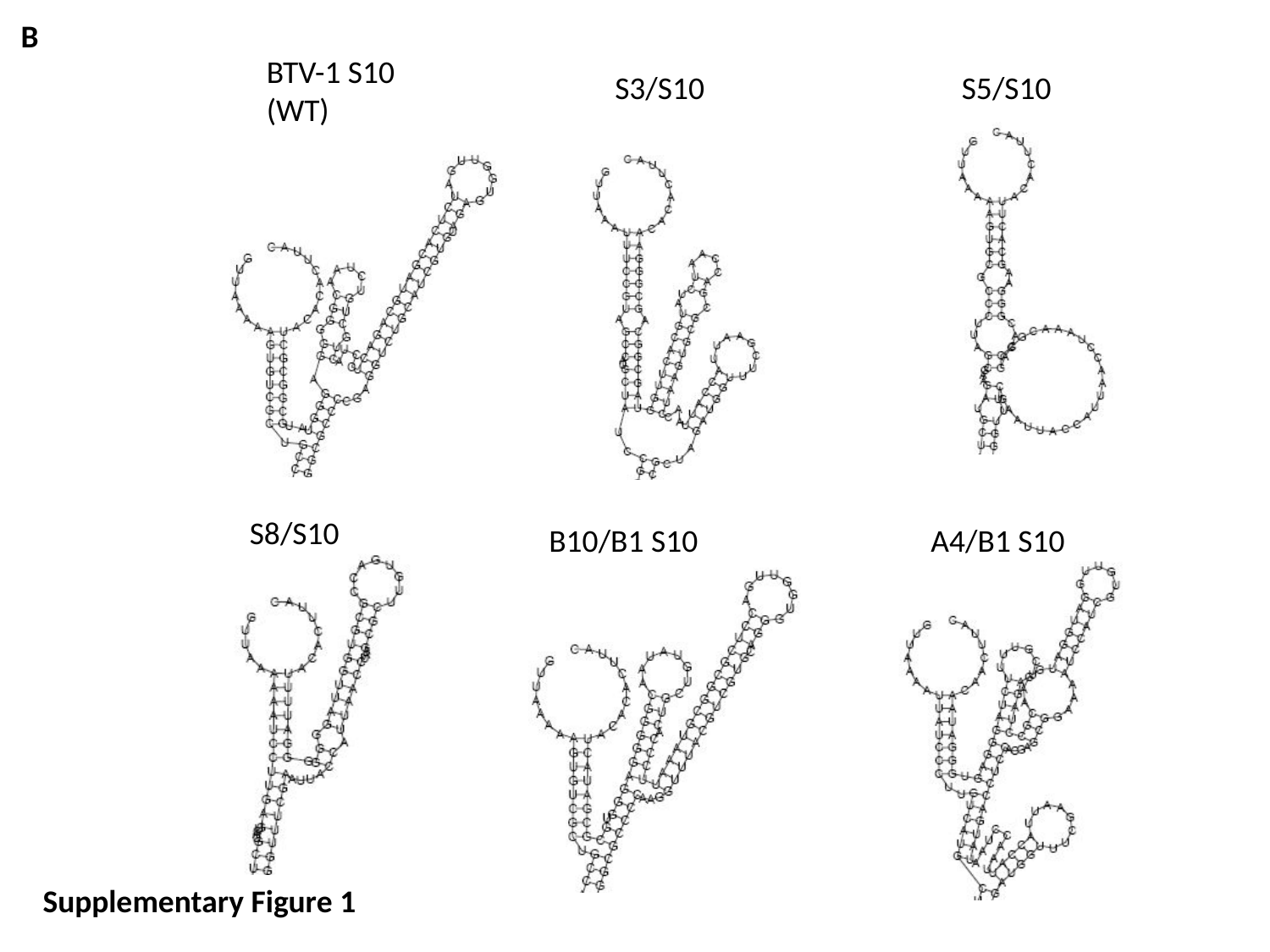

B
BTV-1 S10 (WT)
S3/S10
S5/S10
S8/S10
B10/B1 S10
A4/B1 S10
Supplementary Figure 1

## Slide 3
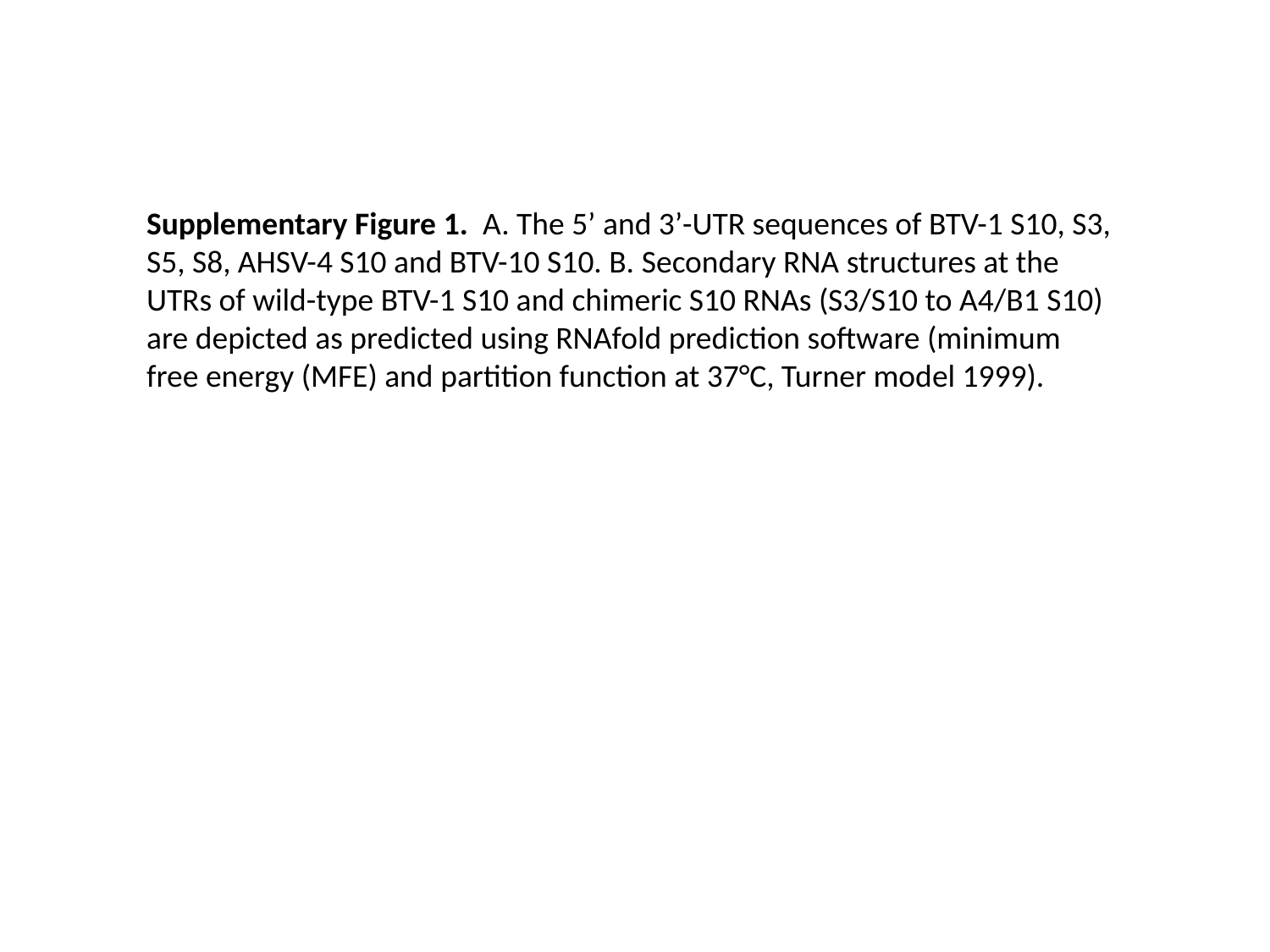

Supplementary Figure 1. A. The 5’ and 3’-UTR sequences of BTV-1 S10, S3, S5, S8, AHSV-4 S10 and BTV-10 S10. B. Secondary RNA structures at the UTRs of wild-type BTV-1 S10 and chimeric S10 RNAs (S3/S10 to A4/B1 S10) are depicted as predicted using RNAfold prediction software (minimum free energy (MFE) and partition function at 37°C, Turner model 1999).
